# Structured Polymer-Derived Ceramic Composites via Near-Infrared Thermal Stereolithography

**DOI:** 10.1021/acsapm.5c00241

**Published:** 2025-07-14

**Authors:** Evelyn Wang, Shruti Gupta, Charles J. Rafalko, Benjamin J. Lear, Michael A. Hickner

**Affiliations:** † Department of Chemical Engineering, 3078 Michigan State University, East Lansing, Michigan 48824, United States; ‡ Department of Material Science and Engineering, 8082 Pennsylvania State University, University Park, Pennsylvania 16802, United States; § Department of Chemistry, Pennsylvania State University, University Park, Pennsylvania 16802, United States

**Keywords:** additive manufacturing, 3D NIR thermal SLA, polymer-derived ceramic, mechanical properties, silicon carbide

## Abstract

We have developed near-infrared (NIR) thermal stereolithography (SLA) to print 2.5D-structured polymer-derived ceramic (PDC) composites with high SiC particle loadings in a PDC matrix. When combined with polymer infiltration and pyrolysis (PIP), this approach overcomes the challenges associated with traditional ultraviolet-based printing techniques when printing composite resins, namely, low light penetration, limited particle loadings, high shrinkage, and weak mechanical properties. Using an NIR laser to deliver spatially controlled thermal energy to the surface of a reactive resin pool induces localized thermally initiated free-radical polymerization in a top-down SLA configuration. After printing the green body, postprocessing methods, including debinding and PIP, are employed to densify and strengthen the printed samples. A Si–O–C_*x*_ support network was formed in the debinded samples using a small amount of preceramic polymer in the printing resin to maintain the structural integrity of this porous preform. After 5 cycles of PIP, the PDC composites demonstrated a flexural strength of 74.3 ± 13.7 MPa with a density of 2.31 g/cm^3^. Different 2.5D lattice designs were fabricated by using this printing and materials processing method, and a compressive strength of 32.8 ± 11.2 MPa was obtained for lightweight honeycomb structures with an effective density of 1.07 g/cm^3^.

## Graphical abstract

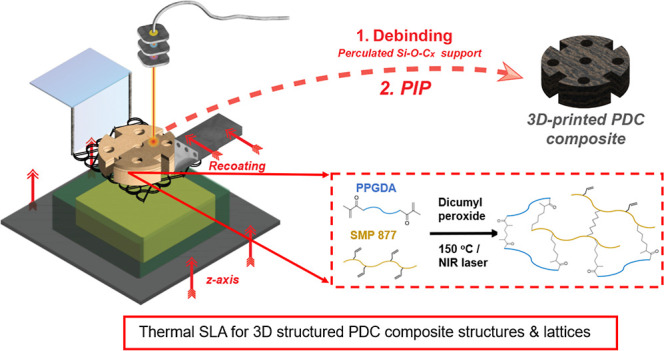

## Introduction

As a high-performance material, ceramic matrix composites (CMCs) are gaining attention for a broad spectrum of applications, including aerospace, biomedical, energy, and electronics.^1–3^ However, relative to other materials, CMCs have achieved a limited role in these applications, primarily because ceramics are difficult to process into intricate structures, which can limit their development.^4^ Despite having excellent mechanical properties, environmental resistance, and temperature tolerance, the hurdles surrounding ceramic processing have primarily limited the adoption of this class of materials.^5^

The introduction of polymer-derived ceramics (PDCs) has opened the door to combining the mechanical performance of CMCs with the superior processability of polymer.^6^ Liquid preceramic polymers (PCPs) can be shaped into a component with complex geometries due to the flexibility offered by polymer processes such as molding and additive manufacturing.^7^ These shaped PCP green bodies can then be pyrolyzed under high temperatures and transformed into ceramics such as SiC, SiOC, SiCN, Si_3_N_4_, and SiBCN, among others.^5,8^ PDCs are used in CMC infiltration, ceramic fiber fabrication, and additive manufacturing (AM).^6^ Unlike traditional ceramic AM with powder processing,^5^ PDCs do not require techniques for consolidating a ceramic structure using traditional sintering pathways,^9^ which makes PDC-AM a potential low-temperature method of producing ceramic parts.^10^ The pyrolysis temperature of PCPs ranges from 800 to 1300 °C,^4^ which is significantly lower than the sintering temperature of ceramics like SiC and Si_3_N_4_.^1^ Moreover, PDCs can offer high modulus, high strength, and oxidation and creep resistance up to 1500 °C, even though they are usually semicrystalline with the presence of crystalline ceramic nanodomains in an amorphous matrix.^11^

The development of AM methods has progressed rapidly, evolving from simple 2D printing techniques into stereolithography (SLA) or additive manufacturing of three-dimensional objects (3D printing).^12^ With the advent of readily available 3D printing hardware, researchers have focused on adapting the well-defined principles of AM to a wide range of materials. It has been reported in the literature that there are various adoptions of 3D printing technologies based on 2D platforms, such as origami-inspired approaches^13^ (2D plane folding into 3D structures), layer-by-layer stacking,^14^ and 1D extrusion direct writing.^15^ Currently, a number of 3D printing methods have achieved maturity—fused filament fabrication, selective laser sintering (SLS), digital light processing (DLP), direct ink writing (DIW), material/binder jetting, and others.^8,10^ Ultraviolet (UV) light-based processes, specifically DLP and SLA, have enjoyed widespread adoption in several manufacturing processes and have been demonstrated to be effective in producing complex and smooth 3D structures with functionalized PCPs.^5^ However, UV-based printing of PCPs is limited in terms of materials and resin compositions. Dense CMCs with high particle loadings are desired for resin compositions. However, the large refractive index mismatch between the filler particles and the polymer precursors inevitably leads to significant light scattering and reduced penetration depth, lowering the printing resolution and decreasing print speed.^16^ Direct UV-SLA of polysilazanes requires a photoinitiator absorbing in the UVC region.^17^ Such short-wavelength UV light is more likely to cause damage to the polymer and requires a high-power mercury-vapor lamp.^18^ Even though many reports add cross-linkers with vinyl,^9^ acrylate,^19^ or thiol^17^ functionality to facilitate the printing of PCP, they inevitably lower the ceramic yield of PDC and increase the carbon content in the pyrolyzed ceramic samples.^20^

Particle-filled resins, including those containing SiC particles, have been demonstrated to increase the density and strength of the PDC samples. Different printing technologies have been adapted for SiC-based resins, including DIW, SLA, DLP, and SLS.^21,22^ While there have been reports on utilizing UV-based SLA/DLP for SiC-based reins, their application has been limited to resins with large particles sizes, which will settle quickly in the resin causing inhomogeneity^23,24^ and require special conditions including long curing times and high energy intensity for printing.^23,24^ We have presented a thermal-SLA method that can facilitate fast printing of micron-size SiC-loaded resins with high particle content.^25^

There have been two demonstrations of harnessing thermal energy in realizing thermal-based 3D printing in the literature as an alternative to UV-based SLA—either directly utilizing the thermal energy from the absorption of near-infrared (NIR) laser for polymerization^26,27^ or using additives like gold nanoparticles as photothermal converters for polymerization.^28^ The most significant advantage of using a NIR thermal SLA to induce thermal curing of the resin is the broad potential scope of thermal curing chemistry across a range of materials.^26^ Also, the NIR thermal SLA technique can print resin compositions with high particle content (47.6 wt % SiC particles (1 μm) in this report), which has not been demonstrated with a UV-based printer due to light penetration issues. Since the high-intensity laser heats the resin rapidly, the cross-linking reaction occurs quickly, with sufficient green strength achieved in as little as a 10th of a second during the printing process. The resulting ceramic parts from thermal SLA have relatively high resolution and smooth surfaces compared to 3D printing methods like DIW.^27^ Finally, postprocessing is simplified for CMCs printed via NIR thermal SLA, where the green body can be pyrolyzed into a ceramic component after simple washing and postcuring, similar to conventional UV SLA post-treatments. We have demonstrated NIR thermal SLA previously for PDC-based particle resins in our previous work.^25^ In this paper, we demonstrate enhanced mechanical properties and higher dimensional accuracy in the fabricated components.

Polymer infiltration and pyrolysis (PIP) is an effective method of obtaining reinforced CMC materials, during which PCP is infiltrated into the porous preform and subsequently pyrolyzed into PDC.^29^ A larger number of PIP cycles can lower the residual porosity in the preform and yield a densified and reinforced structure, with densities approaching the theoretical material density. Repeated PIP cycles (up to 5 or 7 cycles) benefit the sample by providing more linkages between the particles while simultaneously vaporizing unnecessary atoms, thereby increasing ceramic yield and mechanical strength with each successive pyrolysis step.^30^

In this work, we report developing a NIR thermal SLA printing method for the additive manufacture of highly loaded resin compositions. A porous body composed primarily of SiC particles with a supporting PCP-derived structure is fabricated through printing and subsequent debinding. Durazane 1800 was used in multiple cycles of PIP to produce dense PDC composites. The printed samples and lattices demonstrate reasonable flexural strength and compressive strength, which demonstrates that this printing method is capable of producing lightweight particle-based CMCs with excellent mechanical properties and size features on the order of millimeters.

## Experimental Section

### Materials

Poly­(propylene glycol) dimethacrylate (PPGDA, *M*_n_ = 560), acetone (99.5%), and dicumyl peroxide (98%) were purchased from Sigma-Aldrich (St. Louis, MO). Durazane 1800 was supplied by Merck KGaA (Darmstadt, Germany), and silicon carbide (1 μm, β-phase, >99.5%) was purchased from Beantown Chemical (Hudson, NH). SMP 877 resin was purchased from Starfire Systems (Glenville, NY). All chemicals were used as received. The optics for the NIR thermal SLA printer were purchased from Thorlabs Inc. (Newton, NJ), and an 808 nm fiber laser (33 W) was supplied by Lumics (Berlin, Germany) as the thermal SLA laser source. Figure 1 shows the chemical structures of the materials used in this report, while Table S1 lists the resin compositions.

**Figure 1:**
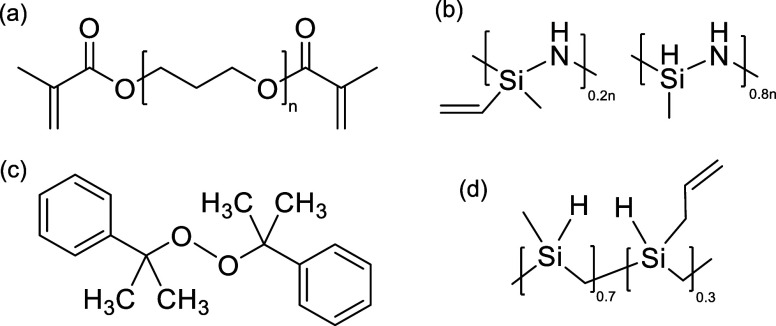
Chemical structures of (a) PPGDA; (b) Durazane 1800; (c) dicumyl peroxide; and (d) SMP 877.

### Sample Preparation

The resin mixture for printing consists of two types of resins: acrylate oligomer PPGDA as the major resin component for cross-linking and facilitating the support of 3D structures and polycarbosilane SMP 877 as the minor resin component for obtaining a percolated Si–O–C_*x*_ supporting structure in the green bodies during debinding. For a typical resin composition, all of the resin ingredients (Table S1) were transferred into a 500 mL round-bottom flask. After adding 100 mL of acetone to the flask with the resin components, the mixture was stirred with a magnetic stirrer at 600 rpm for 12 h. After mixing, the acetone was subsequently removed with a rotary evaporator, obtaining printing resin *PP877*.

### Thermal Stereolithography Printer

The NIR thermal SLA printer consists of four major parts: a high-intensity 808 nm NIR laser fixed onto an optical cage, a fixed build support, a mesh build plate that moves on the *z*-axis, and a resin tray (Figure 2).

**Figure 2:**
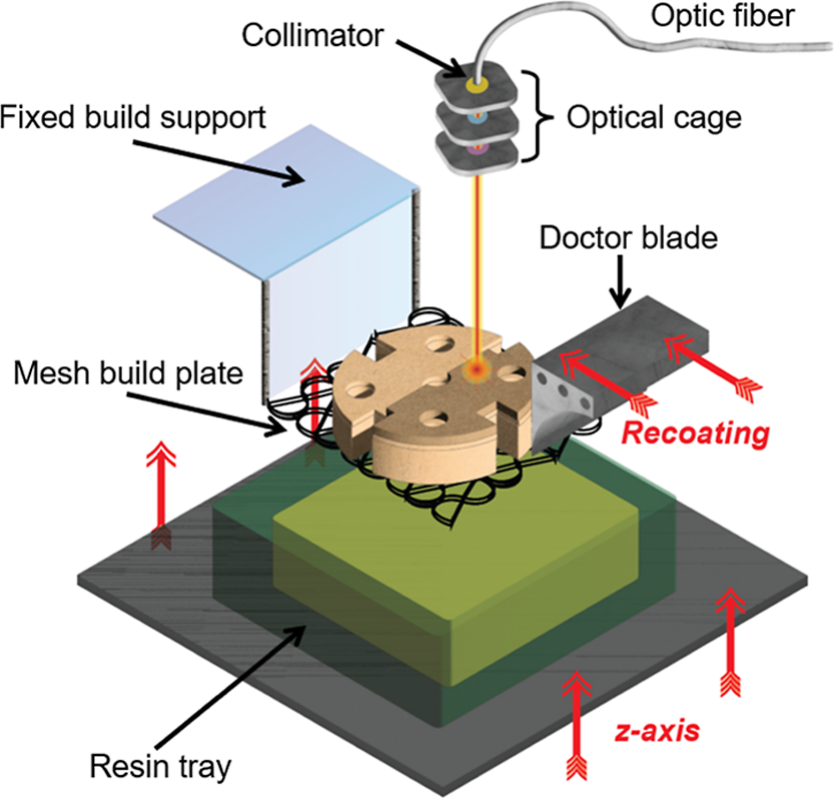
NIR thermal SLA printer for fabrication of 2.5D structures.

The optical cage is attached to an *x*–*y*-axis gantry, where a collimator and a set of lenses are fixed in the optical cage for beam collimation and controlling the laser waist diameter and divergence (eqs S1 and S2). A mesh build plate is fixed onto the build support to hold the newly constructed structures built in a traditional laser-wise fashion. The resin reservoir moves up the *z*-axis to replenish new liquid resin layers onto the printed structures. Notably, the laser and the gantry can move at speeds up to 10 cm/s during printing, resulting in a printing speed comparable to UV SLA.

During a typical printing process, a 3D model is sliced with 3D printing software (Creality Slicer version 4.8.2) to generate a set of G-code instructions for the printer. The 3D printing process begins with immersing the mesh build plate into the resin pool by elevating the resin tray on the *z*-axis support. After the mesh build plate is coated with a single resin layer, the NIR laser writes the first layer onto the high-flow mesh. Then, the resin tray will move up to recoat the resin on the solidified layers with the help of a doctor’s blade, providing new resin layers for the NIR laser to cure. New layers were generated so that a 3D structure could be fabricated through laser and gantry movements. The transition from 2D to 3D printing is achieved by stacking multiple layers together, where each layer contributes to the details and structures of the final prints. Thermal images were taken using a Teledyne FLIR C5 thermal camera (Figure S1), capturing how the NIR laser delivered localized heat to the resin pool. After printing, the parts were washed with acetone 3 times to remove uncured resin, and the samples were fully cured in a vented oven at 150 °C for 20 min.

### Sample Postprocessing

A two-step postprocessing scheme was applied to densify and strengthen the green body. First, the green body was transferred to a vented muffle furnace at room temperature. The sample was subsequently heated to 500 °C with a ramp rate of 0.4 °C/min, dwelled for 1 h, and cooled down to room temperature with a ramp rate of 1 °C/min for decomposition and removal of most of the polymeric species. After debinding, the porous preform was subsequently processed by PIP, where the debinded porous preform was densified by multiple cycles of infiltrating PCP and pyrolysis. The debinded sample was transferred into a three-neck round-bottom flask during a typical PIP cycle. Then, the flask was sealed and degassed until the system reached a pressure of less than 5 kPa. A low-viscosity (20 °C, 10–40 cP) PCP Durazane 1800 was chosen for backfilling the pores/channels in the sample. Durazane 1800 (with 1 wt % dicumyl peroxide as the thermal initiator) was released dropwise onto the sample at a 10 mL/min rate until the sample was fully immersed. The sample was kept under the vacuum for 20 min until no more bubbles were released from the structures. After infiltration, the sample was transferred to a tube furnace under an argon flow (50 cm^3^/min). The sample was heated to 170 °C with a ramp rate of 1.2 °C/min and subsequently heated to 800 °C with a ramp rate of 0.48 °C/min. The sample was held at 800 °C for 1 h before being cooled to room temperature at 1 °C/min. Repeating this procedure of infiltration and pyrolysis will produce samples processed with multiple PIP cycles.

### Characterization

Fourier-transform infrared (FTIR) spectroscopy was performed on a Bruker Vertex 70 IR spectrometer (Billerica, MA) equipped with a liquid nitrogen-cooled midband mercury cadmium telluride detector. Attenuated total reflection with a diamond crystal was used for analyzing the resin compositions and ceramic chemical compositions in the range of 500–2000 cm^–1^. A scanning electron microscope (SEM) (Verios 5 XHR SEM, Waltham, MA) was used to image the microscopic morphology. The samples were fractured to create a flat surface, where the cross-sectional area was used for SEM imaging. An X-ray photoelectron spectroscopy (XPS) (VersaProbe III, Chanhassen, MN) instrument equipped with a monochromatic Al Kα X-ray source (*h*ν = 1486.6 eV) and a concentric hemispherical analyzer^31^ was used in obtaining the elemental and chemical composition of the samples. The sample cross-section was prepared and immediately analyzed to avoid surface oxidation. An Archimedes density measurement apparatus (ASTM B962-17) was used to obtain the samples’ density after PIP. X-ray diffraction (XRD) (X-ray diffractometer, Malvern Panalytical Empyrean, Malvern, United Kingdom) within a 2θ range of 30–75° was used to analyze the samples’ crystallinity and crystalline phases. The mechanical properties were measured with an MTS Criterion 43 (C43.504, Eden Prairie, MN) load frame. Flexural strength (ASTM C1341-13) was measured with an MTS 1 kN S-beam load cell equipped with a three-point bend fixture. Compressive strength was measured with an MTS 20 kN S-beam load cell equipped with compression platens. Thermogravimetric analysis (TGA) was performed with an STA 449 F3 Jupiter (Netzsch, Germany).

The shrinkage of the sample after postprocessing can be calculated with eq 1

1 $$S=\frac{L_{0}-L_{1}}{L_{0}}\times 100\%$$

where *S* represents the linear shrinkage of the sample after debinding and *L*_0_ and *L*_1_ are the lengths of the sample before and after debinding, respectively. The linear shrinkage of the sample after postprocessing is 10.3% (PIP does not change sample dimensions). The flexural strength was measured using an MTS Criterion 43 (C43.504, Eden Prairie, MN) with an MTS 1 kN S-beam load cell in a three-point bend fixture. Flexural strength σ is given by eq 2 (ASTM C1341-13)

2 $$\sigma =\frac{3FL}{2{bd}^{2}}$$

where *F* is the fracture load (N), *L* is the support span length (m), and *b* and *d* are the width (m) and thickness (m) of the sample, respectively. Three samples (*n* = 3) were prepared for both flexural and compression testing. Due to the material’s extremely brittle nature, a test speed of 0.1 mm/min was applied for all tests. Flexural test specimens for three-point bending measured 2 mm in thickness, 6 mm in width, and 45 mm in length and were tested using a support span of 32 mm. The dimensions for the compression samples are provided in Figures 3 and S7.

**Figure 3:**
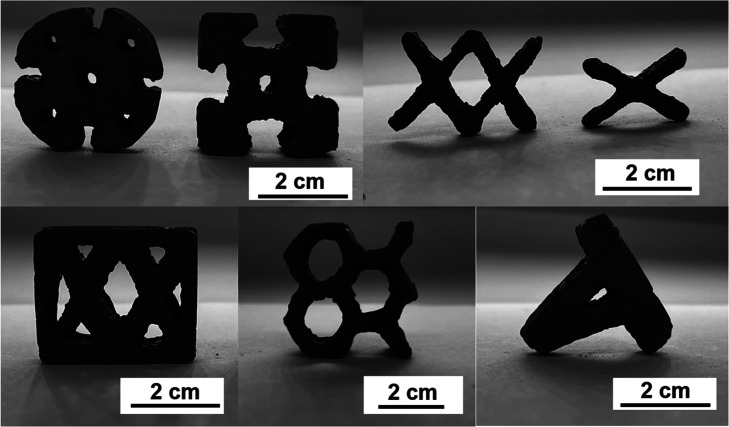
Demonstration of 2.5D-printed green part structures with a NIR thermal SLA printer.

The performance index, *P*_I_, during the compression test is calculated as follows^7^

3 $$P_{I}=\frac{\rho }{\sigma }$$

where ρ and σ represent the sample’s density and compressive strength, respectively. The derivation of eq 3 can be found in Supporting Information.

## Results and Discussion

### Additively Manufactured Parts

The printed 2.5D structures (Figure 3) showed excellent layer-to-layer adhesion with the NIR thermal SLA printer. When hollow 2.5D structures are fabricated, the printer demonstrates the ability to maintain high fidelity and accuracy.

Printing resolution was demonstrated by fabricating different lattice structures, where structures as fine as 1.20 mm can be made through a single scan of the NIR laser. Overall, the NIR thermal SLA demonstrates the capability of reproducing the details from the original 3D models.

### Fourier-Transform Infrared Spectroscopy

FTIR spectra are shown for NIR-printed, uncured printing resin *PP877* (Figure 4a,b); debinded samples with and without Si–O–C_*x*_ structural support (Figure 4c); and debinded green body after different cycles of PIP (Figure 4d).

**Figure 4:**
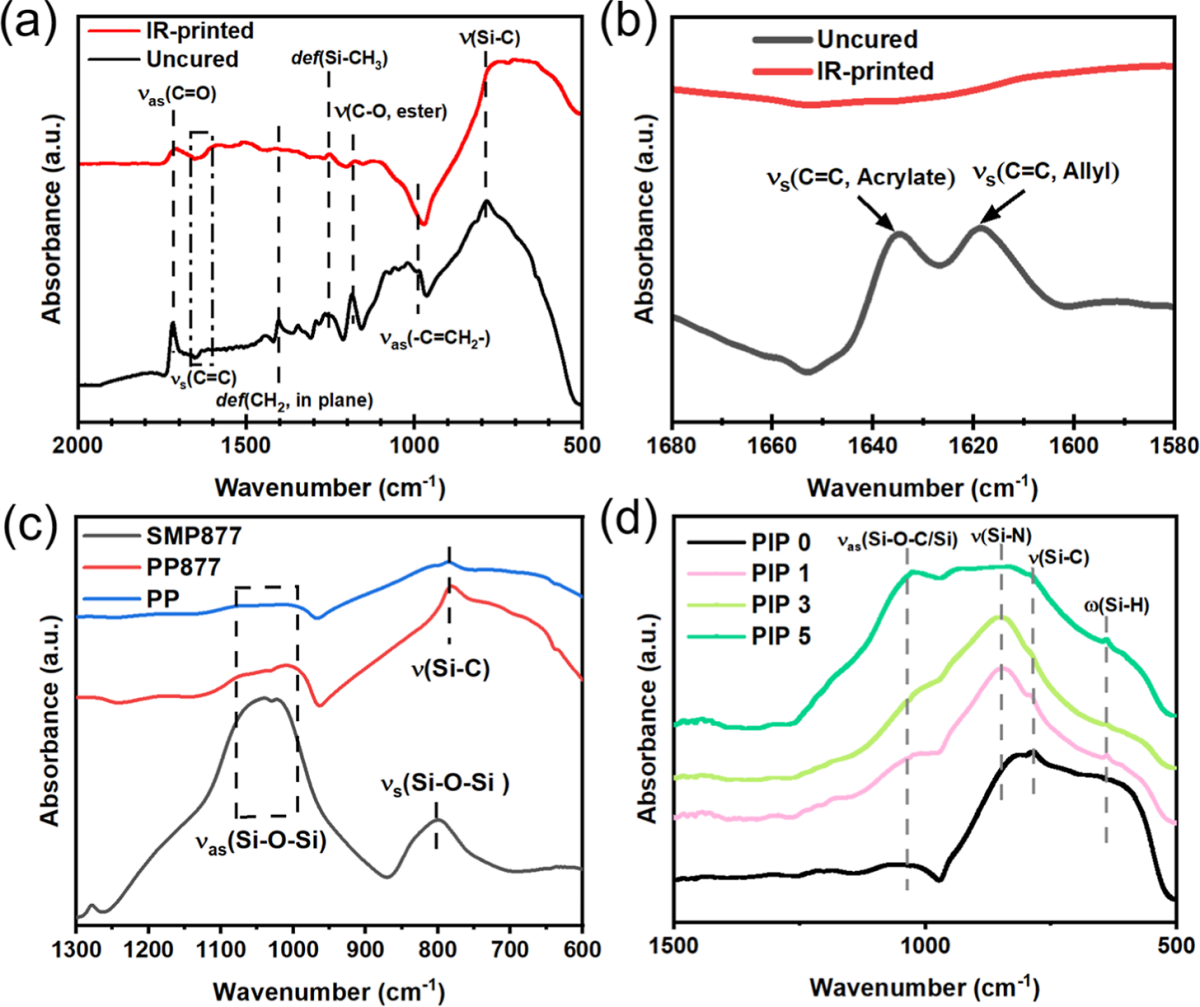
FTIR spectrum of (a) IR-printed and uncured *PP877* samples; (b) IR-printed and uncured *PP877* samples zoomed-in range; (c) SMP 877, *PP*, and *PP877* debinded in the air at 500 °C (in Table S1); and (d) IR-cured *PP877* samples after cycles of PIP.

Figure 4a,b highlights the resin composition before and after IR printing. The diminished ν_as_(–C–H) in the –C=CH_2_– moiety^32^ at 980 cm^–1^ and the disappearance of the acrylic^32^ ν_s_(C=C) and allyl ν_s_(C=C) peaks at 1635 cm^–1^ and 1615 cm^–1^ showed clear evidence of curing of the acrylate-terminated PPGDA and allyl group in SMP 877 resin. While SMP 877 resin does not cure on its own under 200 °C, this data supports that adding the methacrylate resin promotes the cross-linking of the SMP 877 resin. The purpose of adding SMP 877 as PCP before debinding is to support the debinded structure since SMP 877 does not fully burn away (confirmed with TGA, Figure S2). In contrast, pure acrylate resins burn entirely off at this temperature. Figure 4c shows that pure SMP 877 (black curve), after debinding in the air at 500 °C, turns into a network containing a Si–O–Si structure, where the peaks at 1020–1050 cm^–1^ and 780–790 cm^–1^ correspond to asymmetric and symmetric ν­(Si–O–Si),^33^ respectively. Compared to the resin composition with a structural support (*PP877*, red curve), the resin composition without a structural support (*PP*, blue curve) showed a weaker ν­(Si–O–Si) peak. Figure 4d shows the chemical composition of the printed samples after different cycles of PIP, and there is evidence of SiO_2_ ^33^ (ν_as_(Si–O–Si) at 1050–1070 cm^–1^) and SiOC (ν_as_(Si–O–C) at 1025 cm^–1^). Additionally, the ν­(Si–N) bond at 830–840 cm^–1^, ν­(Si–C) bond at 785 cm^–1^, and ω­(Si–H) bond^34^ at 638 cm^–1^ indicate some presence of SiCN and unpyrolyzed Si–H.

### X-ray Photoelectron Spectroscopy

XPS analysis was conducted on the structural support after debinding to determine its chemical composition, Figure 5. Figure S3 shows the XPS analysis of the sample compositions after postprocessing (debinding and PIP).

**Figure 5:**
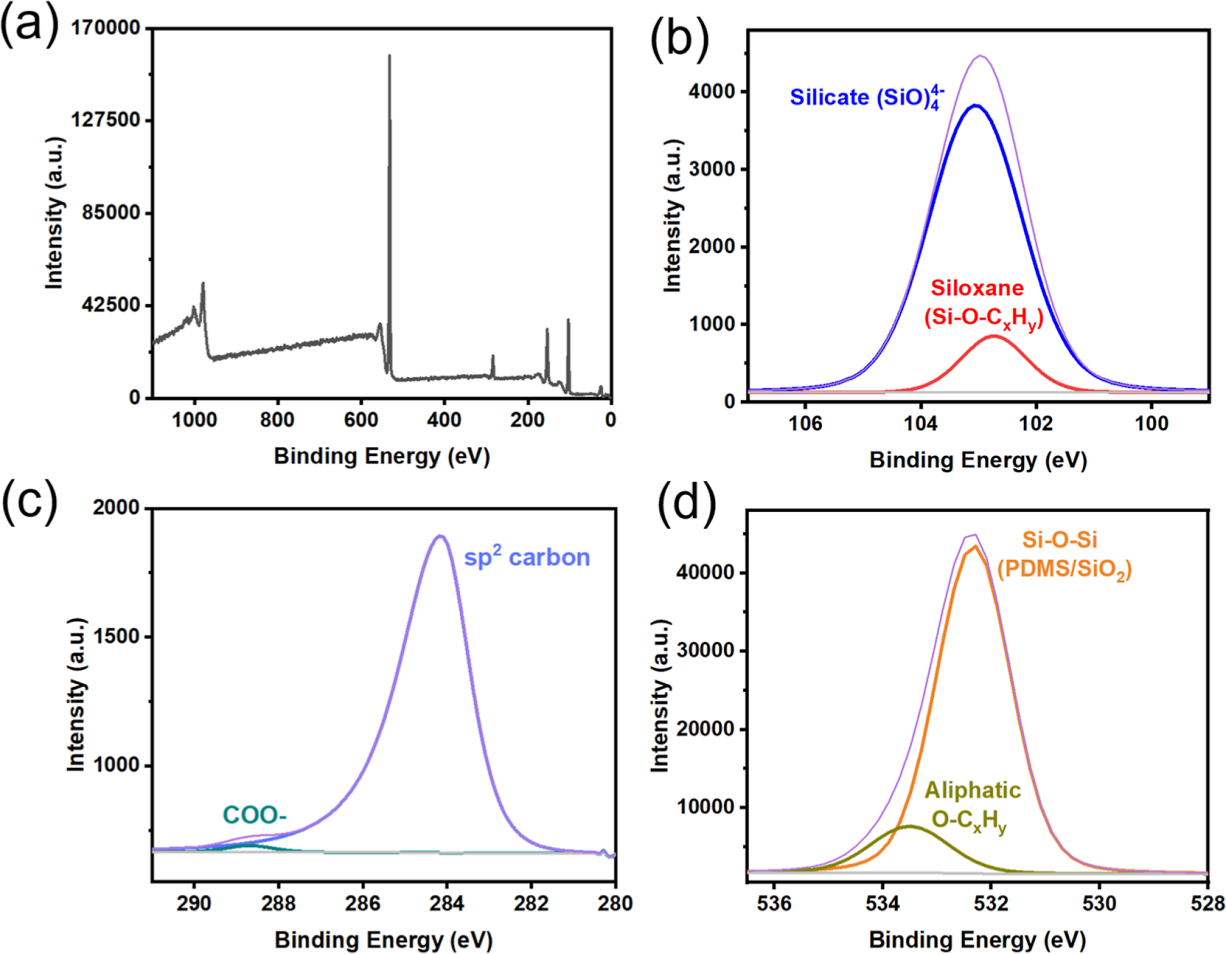
(a) XPS survey spectrum of resin SMP-877 without fillers debinded in the air at 500 °C; (b) XPS spectrum of Si 2p; (c) XPS spectrum of C 1s; and (d) XPS spectrum of O 1s.

Figure 5a shows the survey spectrum of SMP-877 debinded in the air at 500 °C, where the debinding procedure is identical with the green body debinding. In Figure 5b Si 2p spectrum, the XPS peaks at 102.7 and 103.1 eV correspond to siloxane^35^ and silicate,^35^ showcasing both inorganic and organic structural characteristics in the debinded samples. In the C 1s spectrum in Figure 5c, the peaks at 284.2 and 288.7 eV correspond to sp^2^ carbon and COO–, respectively.^35^ The peaks at 532.3 and 533.5 eV (Figure 5d) correspond to Si–O–Si (PDMS/SiO_2_) and aliphatic groups,^35^ reinforcing the previous finding in the Si 2p spectrum that there are both inorganic and organic moieties in the green bodies after debinding in air. Additionally, after debinding in the air at 500 °C, SMP 877 turns into a rigid, yellow-colored solid structure instead of a white powder, which is evidence of the percolated Si–O–C_*x*_ network being formed in the debinded 3D-printed samples.

When combining insights from the FTIR data in Figure 4c, it can be concluded that adding SMP 877 into the green body and debinding in air will turn into a network of Si–O–C_*x*_. Meanwhile, the XPS data (Figure S3) also confirm the existence of SiOC and SiCN in the final sample. Incorporating SiOC and SiCN by the PIP process densifies the porous preforms and strengthens the material.

### X-ray Diffraction

XRD analysis was conducted on the pyrolyzed Durazane 1800 sample and *PP877* sample after 5 cycles of PIP to analyze the crystalline structure and phases of the matrix PDC and the samples after PIP.

It is evident in Figure 6a that there is no distinguishable crystalline species from the pyrolyzed Durazane, where the amorphous halo peaks at around 35–38° and 65–70° represent 3C–SiC. These data demonstrate that the PDC introduced in the PIP process is entirely amorphous. The XRD patterns of the samples after PIP and Durazane 1800 after pyrolysis are shown in Figure 6b. It can be concluded that after several PIP cycles (Figure S4b,c), samples have no difference in their composition, where cubic SiC, from the incorporated particles, is found to be the most abundant crystalline structure in all of the samples. The XRD pattern in Figure S4b,c complements Figure 6, showing that the crystalline structure of the sample after postprocessing remains the same, confirming that the samples throughout different PIP cycles are made of crystalline 3C–SiC particles with minor 6H–SiC and an amorphous PDC matrix from postprocessing PIP.

**Figure 6:**
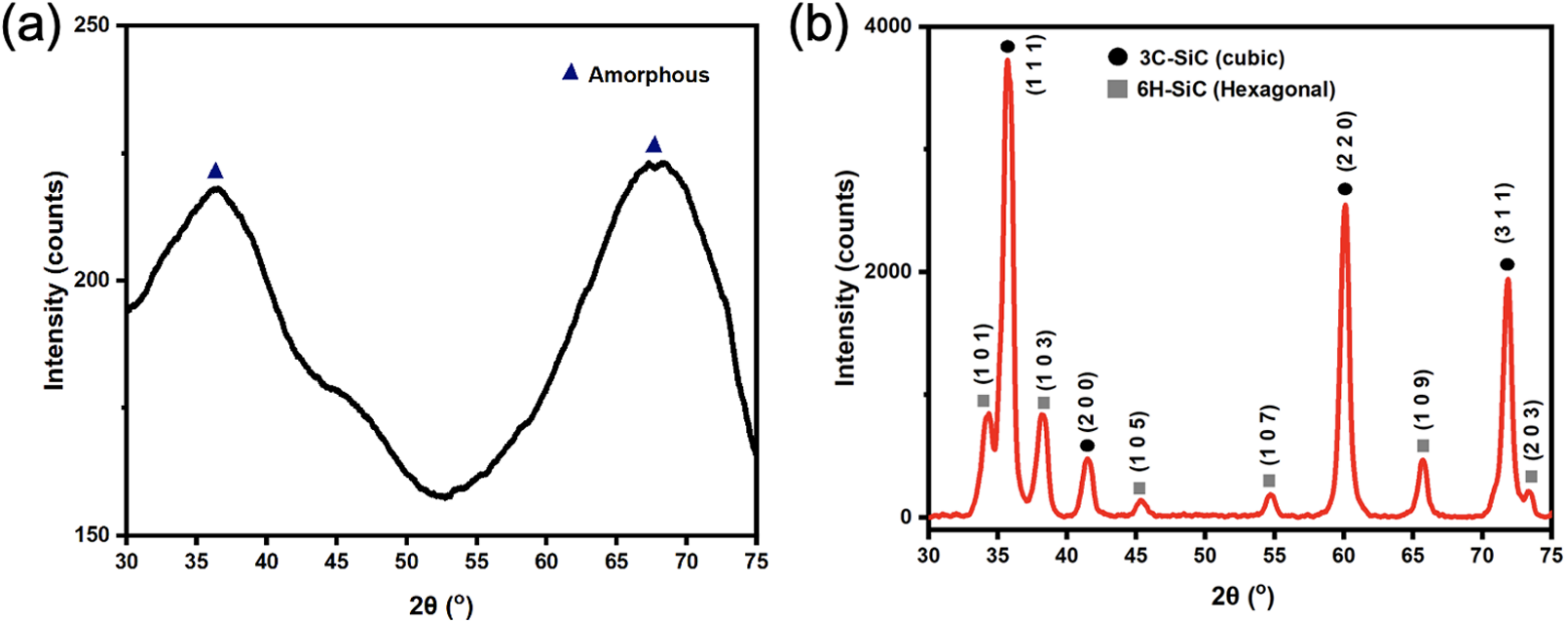
(a) XRD pattern of pyrolyzed Durazane 1800; (b) XRD pattern of the PP877 sample after 5 cycles of PIP. Both samples are pyrolyzed under identical furnace conditions detailed in the Experimental Section.

### Scanning Electron Microscopy

SEM micrographs of debinded samples were taken after different cycles of PIP to analyze structural changes throughout the postprocessing steps. This analysis helps explain the enhanced mechanical properties observed in the 3D-printed samples.

The effects of how PIP strengthens the porous debound samples are shown in Figure 7. From these SEM micrographs, it is evident that with higher PIP cycles, there are reduced openings and cracks throughout the samples. The samples after debinding are composed of SiC particles packed together, and large pores are present in the debinded sample. After 3 cycles of PIP, it is evident that the infiltrated PDC binds the particles together. Most particles are consolidated after 3 cycles of PIP, while the sample still has large openings and cracks. Finally, after 5 cycles of PIP, all the visual cracks and openings are closed, and the particles are entirely bonded with PDC. This composite structure contributes to the high mechanical properties of the samples.

**Figure 7:**
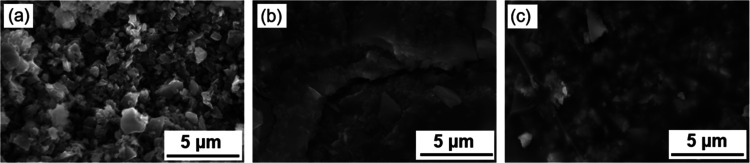
SEM micrographs of the NIR-printed *PP877* sample after (a) debinding (PIP 0, porous preform); (b) 3 cycles of PIP (PIP 3); and (c) 5 cycles of PIP (PIP 5).

It can be concluded that after PIP, the porous preform is impregnated with amorphous PDC, establishing linkages between different particles. Crystalline 3C–SiC particles are bound to each other with amorphous PDC (SiCN and SiOC) from the infiltration and pyrolysis of PCP.

### Mechanical Properties

Fabricating a porous preform will require the removal of the binder in the printed green body. However, binder burnout leaves behind a debinded sample composed of unsintered particles, which have very low mechanical strength and make an intricate component challenging to handle in subsequent processing steps.^36^ For samples with 3D structures and overhanging features, this binder burnout step will weaken the debinded green body (porous preform), and parts with little support or small features will break.^15,37^ Introducing a Si–O–C_*x*_ network in the debinded samples grants the debinded structures (porous forms) enough mechanical strength to support their own weight and arrest cracking during the PIP process.

Figure 8a shows the strength of the porous preforms with and without the Si–O–C_*x*_ support, where *PP* has no support, and *PP877* has the Si–O–C_*x*_ support formed during debinding (detailed compositions are shown in Table S1). Amorphous Si–O–C_*x*_, formed by adding 10 wt % PCP to the printing resin, enhances the flexural strength of the porous preform by as much as 138%. Figure 8b illustrates the relationship between the flexural strength of the samples and the increased number of PIP cycles. Each subsequent PIP cycle increases the density and flexural strength of the CMCs by filling pores and other defects. PIP increases the density of a highly porous debinded sample (PIP 0), where after 5 PIP cycles the density of the sample reaches 2.31 g/cm^3^ (Figure 8c). SEM micrographs (Figure 7) showed 5 PIP cycles completely densifying the sample. The flexural strength of the samples after 5 PIP cycles reached 74.3 ± 13.7 MPa. Representative stress–displacement curves are shown in Figure S6.

**Figure 8:**
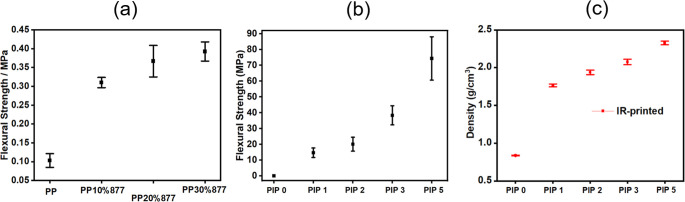
(a) Flexural strength of debinded samples with and without Si–O–C_*x*_ support; (b) flexural strength of NIR-printed *PP877* samples; and (c) density of *PP877* samples throughout different PIP cycles.

### Lattices Compressive Strength

Lattices can have advantages over traditional solid materials,^38^ as these cellular structures are more efficient in achieving excellent mechanical properties with reduced weight.^39^ Vertical stress will lead to a parallel binding force for a solid material to maintain a continuous deformation during compression. This derived binding force will press the material parallelly and cause premature material failure.^40^ Meanwhile, forces redistribute within the structure for the lattice material with hollow structures, making the stress more homogeneous.^39^ The unit cell topology, pattern design, lattice structure, and stress-relieving structures will all affect the compressive strength of the lattice.^38,39^ While there has been research on how lattice properties affect compressive strength, there are still limited methods (e.g., finite element analysis^38,39^ ) for determining the best lattice designs.

Figure 9 is an Ashby plot of compressive strength versus effective density, including porous ceramic materials, as-printed lattices in this report, and solid samples. Porous ceramic materials with different densities will have their characteristic compressive strength. Lattice design will also affect the compressive strength of the samples, where different material designs with the same effective density will have different mechanical properties. A series of lattices, from honeycomb and concentric rings to layered lattices, were tested in this report; see Supporting Information, where the compressive strength of honeycomb lattices is the highest, 32.8 ± 11.2 MPa. The performance index in the Ashby plot is the straight line intersecting the plot, which defines the compressive strength performance under specific densities. Figure 9 shows that the lattices printed in this work have acceptable compressive performance, whereas honeycomb and concentric rings exhibit higher performance above the *P*_I_ line (highlighted line σ/ρ = 10^4^ N m/kg).

**Figure 9:**
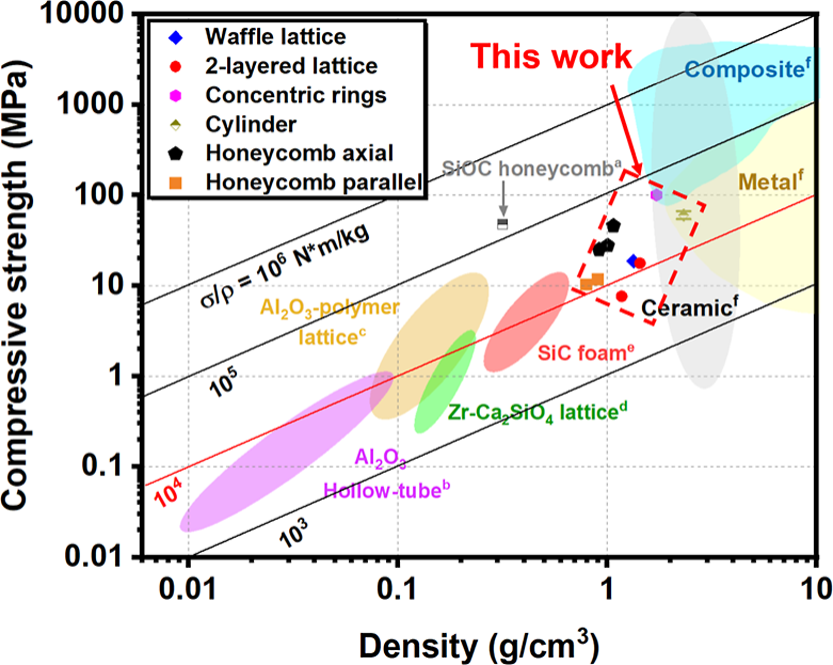
Ashby plot of compressive strength versus effective density for lattice (a,^5^ b,^41^ c,^42^ d,^43^ e,^44^ f^45^). The compression test direction of lattices is shown in Figure S5, lattices are shown in Figures 3 and S7.

## Conclusions

A new NIR thermal SLA fabrication technique is demonstrated in this report. It is shown that with appropriate layer-by-layer adhesion and resin recoating 2.5D-structured high-resolution samples can be made through an NIR thermal SLA printer. This technology can potentially revolutionize the additive manufacturing of PDCs, circumventing the limitations of traditional UV-based SLA. While only a few reports used thermal curing systems with NIR or IR lasers to print PCP materials,^46,47^ we believe this paper proposes a highly versatile NIR thermal SLA printer for the additive manufacturing of 2.5D structures. The printed structures showed a reasonable resolution and smoothness. The introduction of the NIR laser makes it possible to process PCP with high particle loadings and UV-opaque resin compositions. It also has more potential for curing different resins, such as epoxy, polyurethane, PCP, and silicone.

Furthermore, introducing a percolating Si–O–C_*x*_ network in the SiC matrix helps the green body keep its shape after debinding, introducing fewer defects and cracks in the samples. After 5 cycles of PIP, the samples demonstrate enhanced mechanical properties, where the flexural strength of the NIR-printed samples reaches 74.3 ± 13.7 MPa. The compressive strength of the honeycomb lattices was 32.8 ± 11.2 MPa. The compressive strength of lattices printed with a NIR laser lies above the general porous ceramic performance index line (*P*_I_ = 10^4^). Thus, this report’s NIR thermal SLA technique effectively fabricates lightweight PDC composites with enhanced mechanical properties.

## Supplementary Material



## Notes

Laser parameters, performance index derivation, XPS spectra, and XRD patterns of samples after PIP, TGA plot of green body debinding and PDC pyrolysis, compressive strength tests of lattices, thermal images during NIR thermal printing and resin compositions, representative stress–displacement curves, and as-printed lattices (PDF)
