# Supplementary material for: Structured Polymer-Derived Ceramic Composites via Near-Infrared Thermal Stereolithography
Source: ACS Appl Polym Mater. 2025 Jul 14;7(14):8928–36. doi: 10.1021/acsapm.5c00241 (PMC12305487; doi:10.1021/acsapm.5c00241)
Supplement: Supplementary file 1 [file ap5c00241_si_001.pdf]

Supporting information: Structured polymer-derived ceramic (PDC) composites via near infrared (NIR) thermal stereolithography (SLA)

*Evelyn Wang[a], Shruti Gupta[b], Charles J. Rafalko[b], Benjamin J. Lear[c], Michael A. Hickner[a,]**

* Corresponding Author: Michael A. Hickner, Department of Chemical Engineering and Materials Science, Michigan State University, 428 S Shaw Ln, Rm 2258, East Lansing, MI, 48824. E-mail: mhickner@msu.edu

AUTHOR ADDRESS

a: Department of Chemical Engineering, Michigan State University, East Lansing, Michigan, 48824, United States

b: Department of Material Science and Engineering, Pennsylvania State University, University Park, Pennsylvania, 16801, United States

c: Department of Chemistry, Pennsylvania State University, University Park, Pennsylvania, 16801, United States

Laser and printer parameters

The laser's energy density is defined by eq. S1:

$$\eta = \frac{P}{D \cdot v} \quad \text{(S1)}$$

Where η represents the energy density of the laser, P is the optic power of the laser, D is the beam diameter, and ν denotes the laser scanning speed. The laser output was 4.0 W, the laser beam diameter was 1.2 mm, and the scanning speed was 10 mm/s. The calculated energy density of the laser was 33.3 J/cm^2.

The optical system of the NIR thermal SLA printer comprises a laser collimator and two lenses to align and control the beam. Firstly, the collimator collimates the beam scattered in the fiber optic. Beam expanders adjust the beam waist diameter (h) and divergence angle—larger waist diameters reduce divergence. The desired configuration is achieved by placing two lenses at a distance equal to the sum of their focal lengths, eq. S2.

$$\frac{f_1}{f_2} = \frac{h_1}{h_2} = M \quad \text{(S2)}$$

Where f_1 and f_2 are the focal lengths of two lenses, respectively, and M is the magnification level. In the report, two lenses with f_1 = 4 mm and f_2 = 15 mm were used to adjust the distance between the sample and the laser optic. This ensures that there is enough printing area, and that enough distance is maintained between the optic and resin pool (60 mm), so the fumes and resin heating do not damage the optics.

Thermal imaging

A thermal camera was attached to the NIR thermal printer during the 3D printing process, and digital thermal imaging was taken during a typical printing process, as shown in Figure S1.

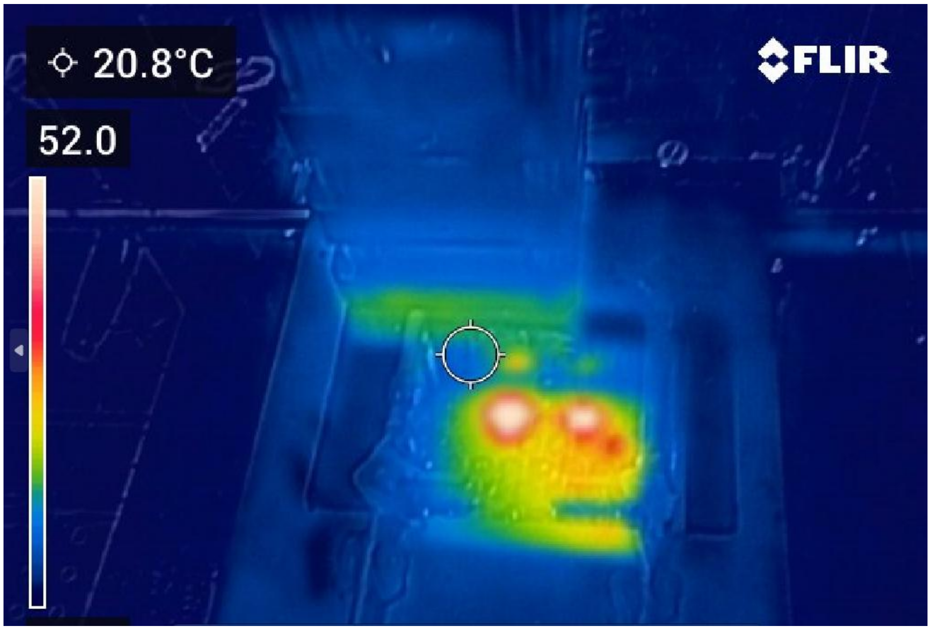

Figure S1. NIR-assisted SLA process under a thermal camera.

Figure S1 demonstrates a typical NIR-assisted SLA process visualized using a thermal camera. Heat is spatially distributed only in the printing area to obtain the desired structures and minimize the thermal curing of non-printed areas.

Resin compositions

Table S1. Resin chemical compositions

	PP	*PP10%877* (printing resin, *PP877*)	*PP20%877*	*PP30%877*
PPGDA / g	10	10	10	10
Dicumyl Peroxide / g	0.1	0.1	0.1	0.1
SMP 877 / g	0	1	2	3
SiC / g	10	10	10	10

Thermogravimetric analysis (TGA)

Thermogravimetric analysis (TGA) was conducted on the *PP877* sample to study the debinding process in the air and on Durazane 1800 samples to analyze the decomposition behavior of the infiltrating PCP.

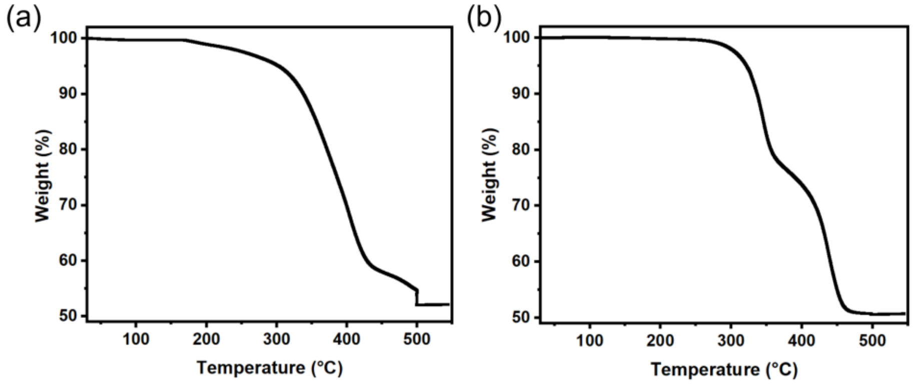

Figure S2. (a) TGA plot of *PP877* heating in the air; (b) TGA plot of Durazane 1800 heating in argon.

The TGA for the *PP877* sample was conducted in air with a heating rate of 10 ^oC/min from room temperature to 500 ^oC. Then, the sample was held at 500 ^oC for 1 h before cooling back to room temperature at the same rate of 10 ^oC/min. The TGA analysis for Durazane was performed under an argon atmosphere (50 cm^3/min), where the sample was heated to 800 ^oC at a heating rate of 10 ^oC/min before cooling down to room temperature with the same ramp rate. From Figure S2(a), it can be concluded that during the debinding of the *PP877* samples, since SiC particles do not react at 500 ^oC, most of the polymeric species are burnt away, leaving behind 2.04 wt%. While acrylate resin completely burns off during the stage, this residual arises from the Si-O-C_x structural support. Figure S2(b) shows that Durazane 1800, the infiltration PCP during PIP, has a ceramic yield of 73.5 wt% when heating to 800 ^oC. Furthermore, the TGA curve demonstrates no major decomposition temperature stages, which points out that there is no need for a dwell stage during PCP pyrolysis.

X-ray photoelectron spectroscopy (XPS) of PIPed samples

XPS analysis was performed on NIR-printed *PP877* samples after debinding and 5 cycles of PIP, as shown in Figure S3. XPS spectra show the chemical composition of the PDC composites after post-processing.

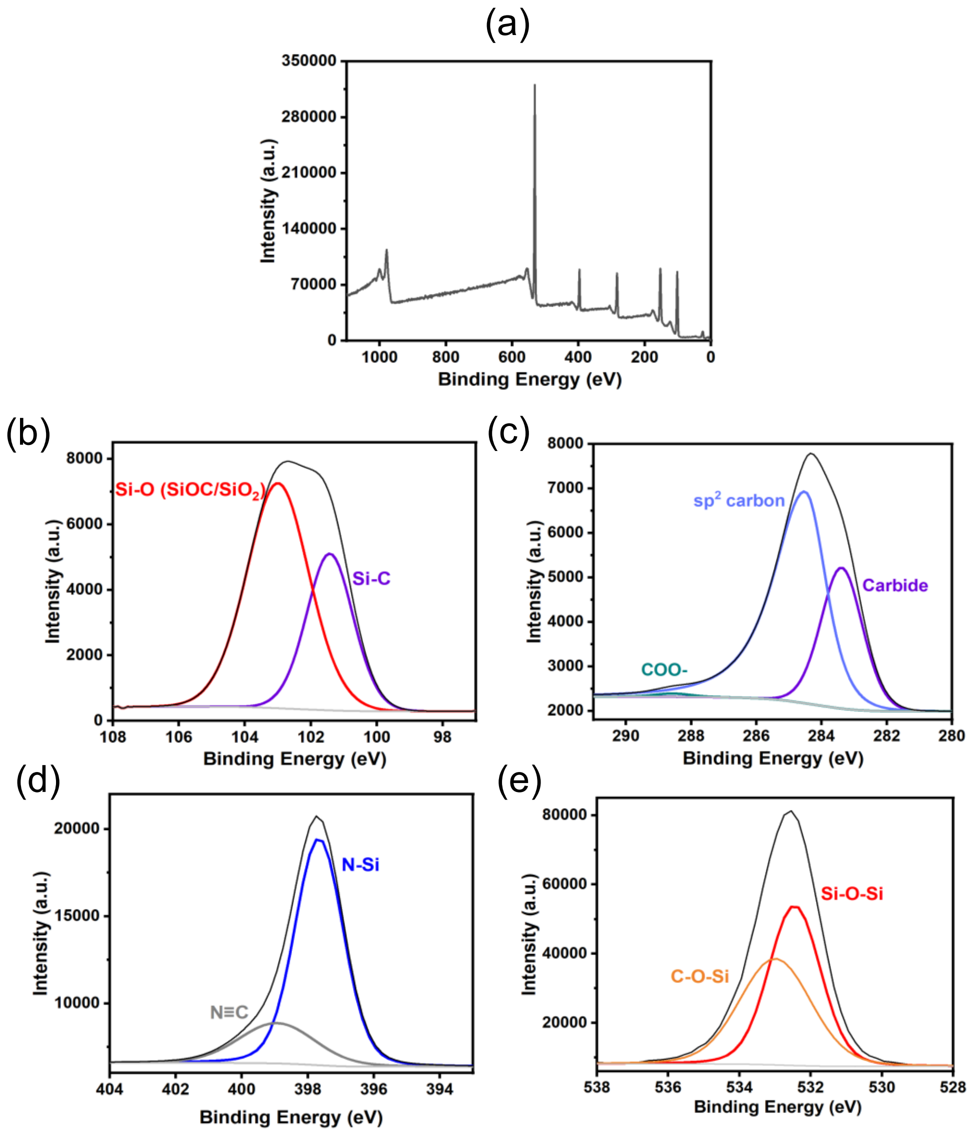

Figure S3. (a) XPS survey spectrum of the IR-printed samples after 5 cycles of PIP; (b) XPS spectrum of Si 2p; (c) XPS spectrum of C 1s; (d) XPS spectrum of N 1s; (e) XPS spectrum of O 1s.

From Figure S3(b) Si 2p spectrum, the peak at 101.5 eV corresponds to Si-C while the peak at 103.0 eV stands for Si-O (SiOC/SiO_2). For Figure S3(c) C 1s spectrum, the peaks at 283.4 eV, 284.6 eV, and 288.9 eV are assigned to carbide (SiC), sp^2 carbon, and COO- bands,

respectively. From Figure S3(d) N 1s spectrum, the peak at 397.7 eV correlates to N-Si, and 399.0 eV is a characteristic peak of N-C. Finally, in Figure S3(e) O 1s spectrum, the two O peaks are for O-Si (532.6 eV) and O-Si-O (534.0 eV).[1]

X-ray diffraction（XRD) of PIPed samples

XRD analysis was conducted on IR-printed samples after 1 and 3 cycles of PIP and commercial SiC particles to analyze the crystalline structure of the samples after post-processing.

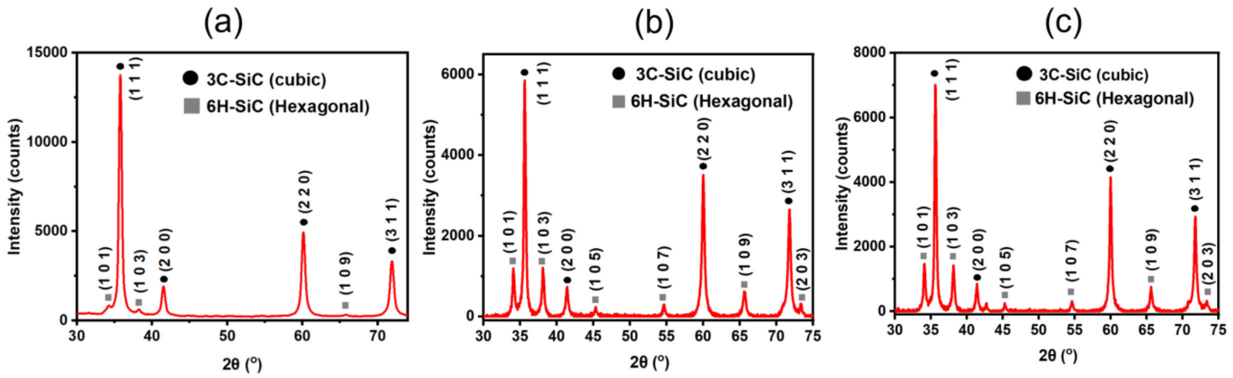

Figure S4. XRD pattern of (a) as-purchased commercial SiC particles; (b) NIR-printed *PP877* sample after 1 cycle of PIP; (c) NIR-printed *PP877* sample after 3 cycles of PIP. All the samples are pyrolyzed under the same furnace conditions mentioned in the experimental section.

Figure S4(a) shows the crystalline structure of commercial SiC particles used in the *PP877* resin composition, where the commercial SiC particles are mainly made of cubic 3C-SiC with a minor hexagonal 6H-SiC component. It can be concluded from Figure S4(b) and (c) that different PIP cycles do not influence the crystalline structure of the samples.

Performance index P_I derivation

Performance index P_I can be derived from eq. S3:

$$\omega = LP\left(\frac{\rho}{\sigma}\right) \tag{S3}$$

Where ω, L, ρ, and σ represent the sample's weight, length, density, and compressive strength. P stands for the pressure applied to the sample upon failure.

Compressive strength

Figure S5 shows the direction in which the compression tests are performed in Figure 9 Ashby plot.

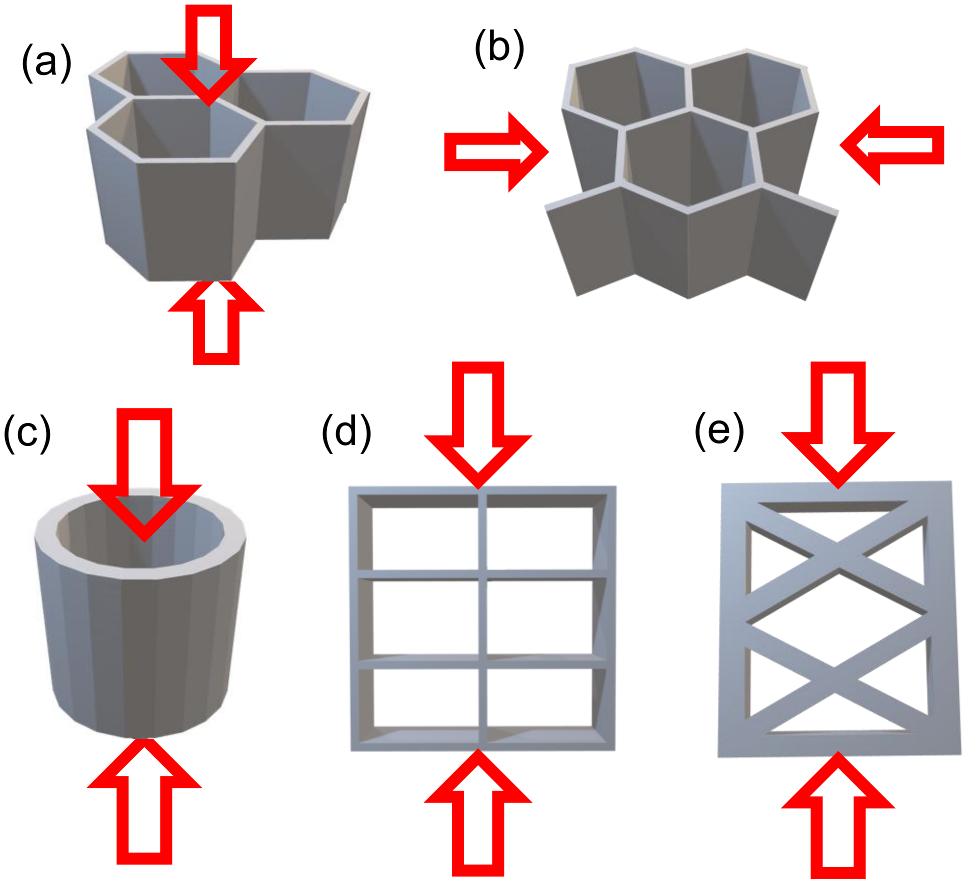

Figure S5. (a) Axial compression of honeycomb structure; (b) Parallel compression of honeycomb structure; (c) Compression of Concentric rings; (d) Compression of a waffle lattice; (e) Compression of a 2-layered lattice.

Representative load-displacement curves are shown in Figure S6.

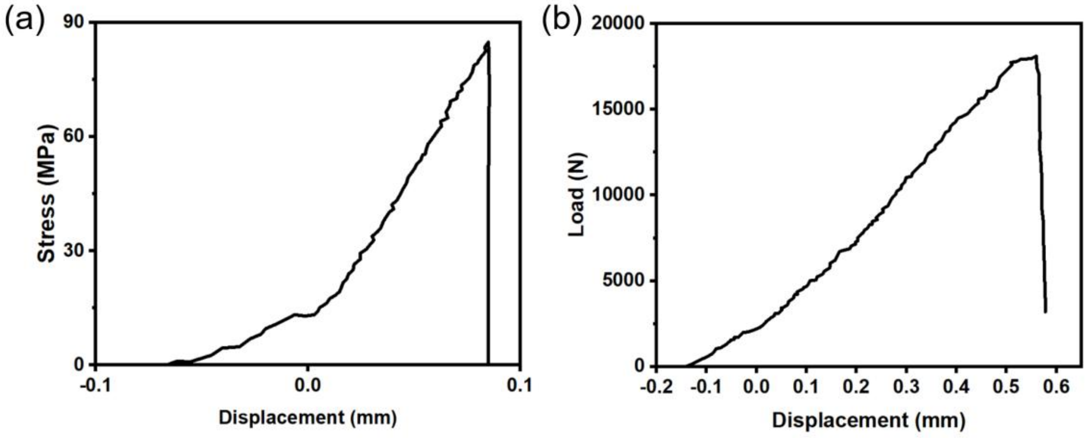

Figure S6. (a) representative stress – displacement curve of *PP877* samples after 5 cycles of PIP during three-point bending flexural strength testing; (b) representative load – displacement curve of *PP877* samples after 5 cycles of PIP during compressive strength testing.

The as-printed lattices from the thermal SLA printer are shown below in Figure S7.

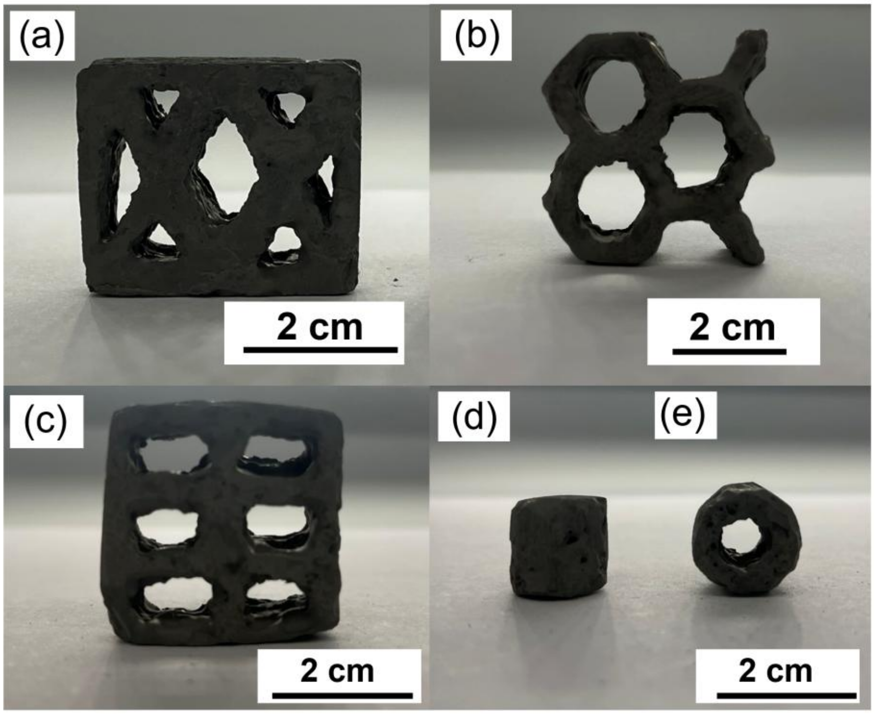

Figure S7. As-printed lattices for (a) 2-layered lattice; (b) honeycomb lattice; (c) waffle lattice; (d) cylinder; (e) concentric ring lattice.

Reference

(1) Abass, M. A.; Syed, A. A.; Gervais, C.; Singh, G. Synthesis and Electrochemical Performance of a Polymer-Derived Silicon Oxycarbide/Boron Nitride Nanotube Composite. *RSC Adv* **2017**, *7* (35), 21576–21584. https://doi.org/10.1039/c7ra01545c.
